# The Evolutionary Pattern of Glycosylation Sites in Influenza Virus (H5N1) Hemagglutinin and Neuraminidase

**DOI:** 10.1371/journal.pone.0049224

**Published:** 2012-11-01

**Authors:** Wentian Chen, Yaogang Zhong, Yannan Qin, Shisheng Sun, Zheng Li

**Affiliations:** 1 Laboratory for Functional Glycomics, College of Life Sciences, Northwest University, Xi'an, People's Republic of China; 2 Department of Pathology, Clinical Chemistry Division, Johns Hopkins University, Baltimore, Maryland, United States of America; University of Ottawa, Canada

## Abstract

Two glycoproteins, hemagglutinin (HA) and neuraminidase (NA), on the surface of influenza viruses play crucial roles in transfaunation, membrane fusion and the release of progeny virions. To explore the distribution of N-glycosylation sites (glycosites) in these two glycoproteins, we collected and aligned the amino acid sequences of all the HA and NA subtypes. Two glycosites were located at HA0 cleavage sites and fusion peptides and were strikingly conserved in all HA subtypes, while the remaining glycosites were unique to their subtypes. Two to four conserved glycosites were found in the stalk domain of NA, but these are affected by the deletion of specific stalk domain sequences. Another highly conserved glycosite appeared at the top center of tetrameric global domain, while the others glycosites were distributed around the global domain. Here we present a detailed investigation of the distribution and the evolutionary pattern of the glycosites in the envelope glycoproteins of IVs, and further focus on the H5N1 virus and conclude that the glycosites in H5N1 have become more complicated in HA and less influential in NA in the last five years.

## Introduction

Influenza A viruses (IVs), which belong to the orthomyxoviridae family, consist of eight negative RNA strands. Hemagglutinin (HA) and neuraminidase (NA) are two glycoproteins that are encoded by the IV genome, expressed from segments 4 and 6, respectively. The selection due to various host immune systems and anti-flu drugs accelerate the mutation rates of viral proteins, especially for these two membrane proteins [1], [2]. There are 17 HA subtypes and 10 NA subtypes, designated H1-H17 and N1-N10, respectively. Over 118 combinations of IVs can be isolated from wild birds, which are also the natural reservoir of these viruses (except the H17N10 virus, which, until recently, was isolated only from bat) [3]–[5]. The species jumping ability of IVs can result in the infections of poultry and mammals, such as chicken, swine, equine or whale species, with different virulence levels [6]–[9]. The H1N1, H2N2 and H3N2 viruses have been responsible for tens of millions deaths during the deadly history of human influenza epidemics. Furthermore, the H5N1, H7N7, H7N2, H7N3 and H9N2 viruses have been isolated from sporadic human infections and deaths [10]–[13]. It is worth noting that the H5N1 virus is the most severe for human and avian species, with sudden onset and high mortality. The mortality rate in hundreds of patients who were hospitalized for H5N1 infections was roughly 59.05%, much higher than the mortality rates of the Spanish Flu or the 2009 influenza pandemic (H1N1) [11], [14], [15].

As a requirement for infection, the homotrimeric HAs play a key role in binding to the host sialic acid (SA) receptors and membrane fusion. The nascent HA of all subtypes consists of conserved structures, including the signal peptide, the cytoplasm domain, the transmembrane domain and the extracellular domain [16]. The mature HA monomer can be cleaved by proteases into the global HA1 and stalk HA2 subunits [17]. When IVs are located in the host digestive tract or respiratory tract cell, the receptor binding domains (RBDs) at the tip of HA1 bind to the SAα2-3Gal or SAα2-6Gal receptors, which are essential for endocytosis [18], [19]. HA unfold and expose the interior HA2 subunits in the acid environment, then the fusion peptides in HA2 insert themselves into the host membrane (viral membrane fusion) [20], [21].

Homotetrameric NA is a type II membrane protein, whereas HA is a type I membrane protein. The nascent NA consists of four parts: the cytoplasm tail (in amino-terminus), the transmembrane domain, the stalk domain and the global domain [22]. Different subtypes of NA are composed of 450∼480 amino acids, displaying low sequence similarity. Although there is variable homology among the various NA subtype sequences, especially in the N1 and N2 subtypes with the deletion of 4∼30 amino acids in the stalk domain [23], NA subtypes display stable topologies: a six-bladed β-propeller fold makes an enzymatic activity domain that functions in the release of progeny virions [24], [25].

**Figure 1:**
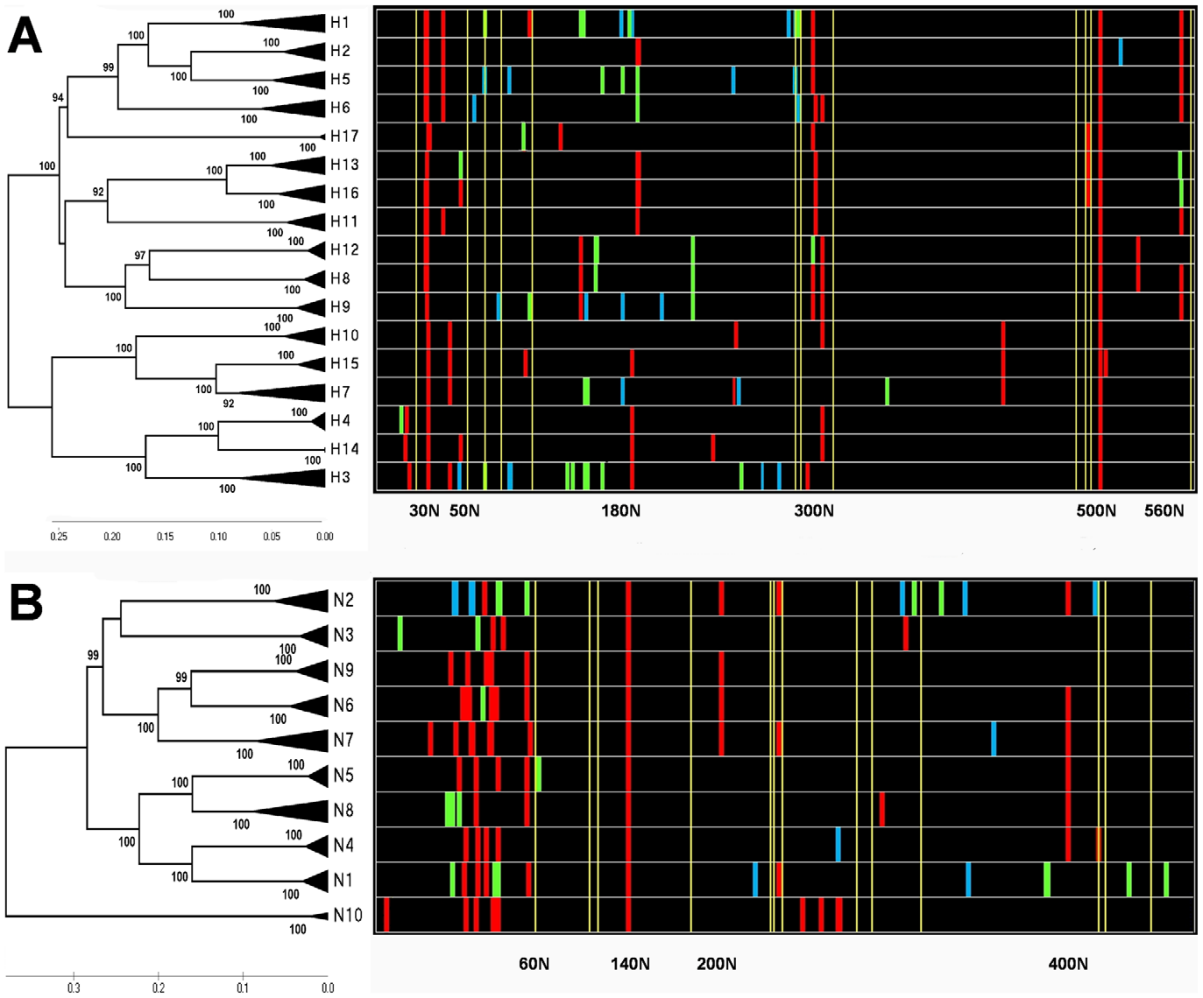
The N-J trees of two glycoproteins in IVs with the corresponding distribution chart of glycosites. The phylogenetic trees of HAs and NAs were constructed using three to ten representative amino acid sequences in each subtype (File S1). The distribution charts of glycosites, colored according to the statistics of conservation in each HA or NA subtype (File S2), are shown in various strips. The red, green and blue color represent the levels of conservation of “>95%”, “5%∼95%” and “<5%”, respectively. The conserved cysteines are shown in yellow strips. (A) The N-J tree of HA subtypes with the corresponding distribution chart of glycosites. (B) The N-J tree of NA subtypes with the corresponding distribution chart of glycosites.

HA and NA have a distribution ratio of 4∶1 on the influenza viral envelop and maintain the basic functions of host recognition, infection and viral diffusion [26]. Various studies have reported that some of the factors that influence HA include the number of the basic residues in the HA0 cleavage site, the mutation of key residues in the RBD, the changing of antigenic sites or N-glycosylation sites (glycosites) and the variation of the topology of N-glycan structures [15], [27]. Meanwhile, the factors that influence NA include deletion of the stalk domain, the mutations drug-resistance, as well as the changing of antigen sites or glycosites and the variation of the topology of N-glycan structures [28]. As a kind of glycan-binding protein (GBP), HA works with NA, which functions as an exoglycosidase, cooperatively. Only by achieving a dynamic equilibrium between attaching to the host and releasing progeny virions can the IVs gain a long-term mechanism for infection and diffusion.

**Figure 2:**
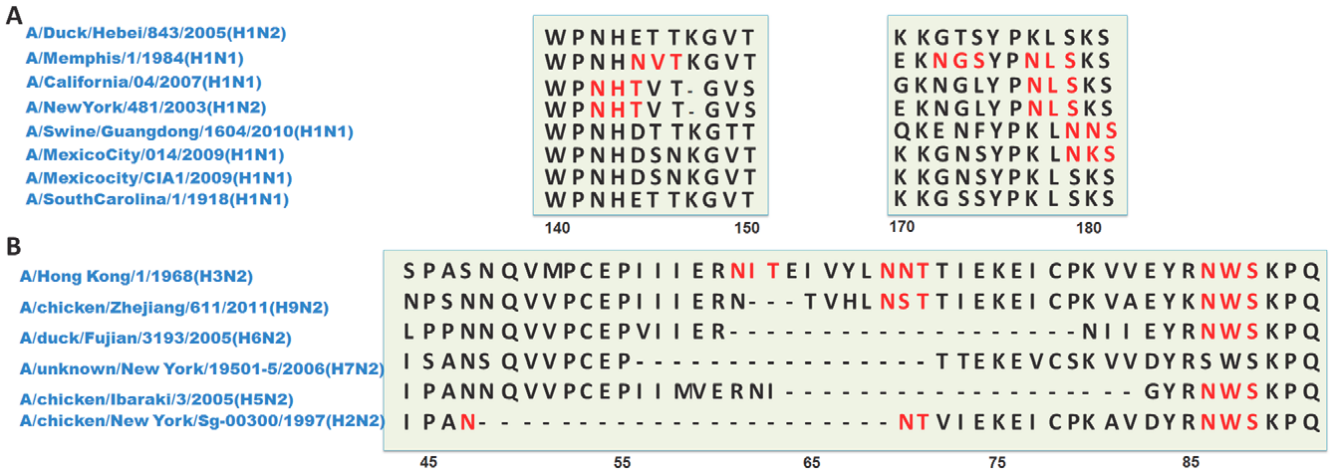
The alignment of glycosylated regions in H1 HA and N2 NA sequences. (A) Most adjacent glycosites in one region were determined by antigenic drift, compared to a fraction of nearby glycosites that were caused by antigen shift. Although the 144NVT/142NHT glycosites are common in seasonal H1N1 viruses, they have different origins. The 177NLS/179NKS glycosites were another common feature in the H1N1 virus. Obviously, the seasonal IVs with long-term circulation had more glycosites than the avian flu or pandemic flu. (B) The pattern of stalk domain deletion in N2 NA is distinctive in different combinations of IVs. The representative NA sequences in each available N2 subtypes are aligned in the stalk domain. As can be observed, the number of deletions varies from 4 to 24 residues, causing the loss of one or two potential glycosites. Among these, a new glycosite emerged in the restructured NA of the avian H2N2 virus.

The existence of N-glycosylation is necessary for viral membrane glycoproteins. The biosynthesis and modification of nascent secretory or membrane proteins occurs in the endoplasmic reticulum and Golgi, N-linked glycans encode crucial information for the folding, maturation, transport or degradation of proteins [29]. To escape both the host's humoral and cellular immune systems, the potential glycosites in viral envelope proteins can provide the identical glycans as those of the host's cells to mask the antigenic sites [28], [30]. Additionally, glycosylation also impacts the sensitivity of HA to temperature, the protection of cleavage sites and the stalk domain, and even the receptor-binding preferences [31]–[33]. As the ideal model for the influence of N-glycosylation in pathogen-host interaction, the present studies show that the envelope glycoproteins of IVs appear to only have N-glycosylation, with no O-glycosylation and GPI-anchors [34]; hence, the glycosites discussed in this paper only pertain to N-glycosylation site.

**Figure 3:**
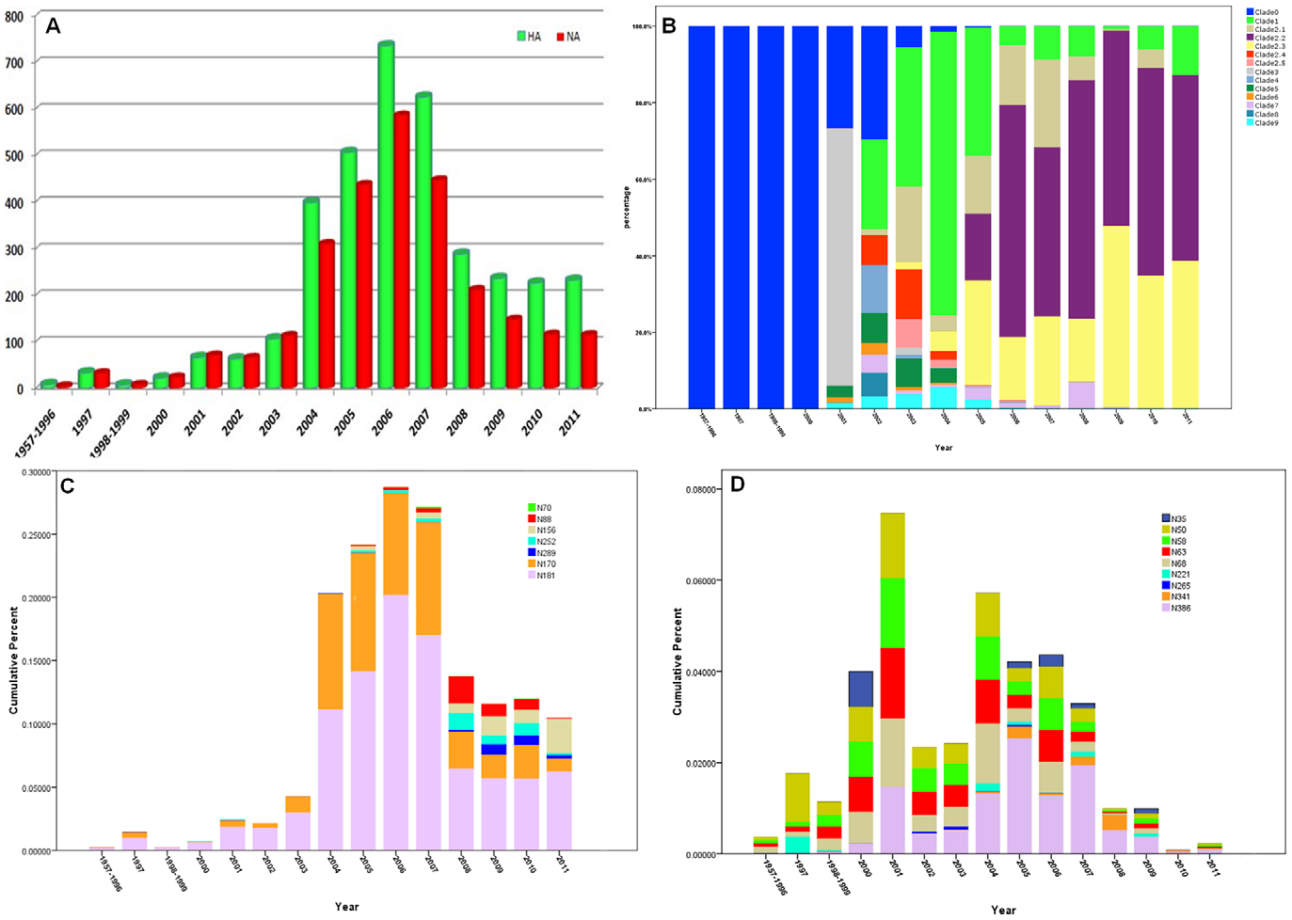
The distribution regularity of glycosites in different H5N1 clades. The statistical analysis in HA and NA indicated that the glycosites in H5N1 have become more complicated in HA and less influential in NA in the last five years. All the sequences of HA and NA were contained in File S3 and S4. As is shown, the records in 1957∼1996, 1998∼1999 and 2012 were so limited that should be combined or exclueded. (A) The recording numbers of HA and NA in recent years. The ealiest H5N1 virus record first appeared in 1957 and increased rapidly after 2004. (B) The percentage of various clades or subclades from 1957 to 2011. The early H5N1 viruses belonged to clade 0 and diversified after 2002. The most common clade 2.2 and 2.3 became dominant after 2008. (C) The cumulative percent of unconserved glycosites in HA. The evolution of six unconserved glycosites showed the diversity of N-glycosylation has become common after 2007. (D) The cumulative percent of unconserved glycosites in NA. The evolution of four conserved glycosites in the stalk domain and five unconserved glycosites showed the frequence and diversity have falled rapidly after 2007.

Current studies have analyzed the evolutionary dynamics of N-glycosylation sites of select subtypes or HA/NA as a whole [35]–[37]. Although most influenza evolution can be accounted for by genetic drift, there is also evidence of adaptive evolution of mutations which are under positive selection [38], [39]. We have extended previous studies of basic similarity alignment scoring of similarity to analyze the position-specific glycosites that are under selective pressure in IVs [40]. Here we present a detailed investigation of the distribution and the evolutionary pattern of the glycosites in the envelope glycoproteins of IVs, especially in the H5N1 virus.

**Figure 4:**
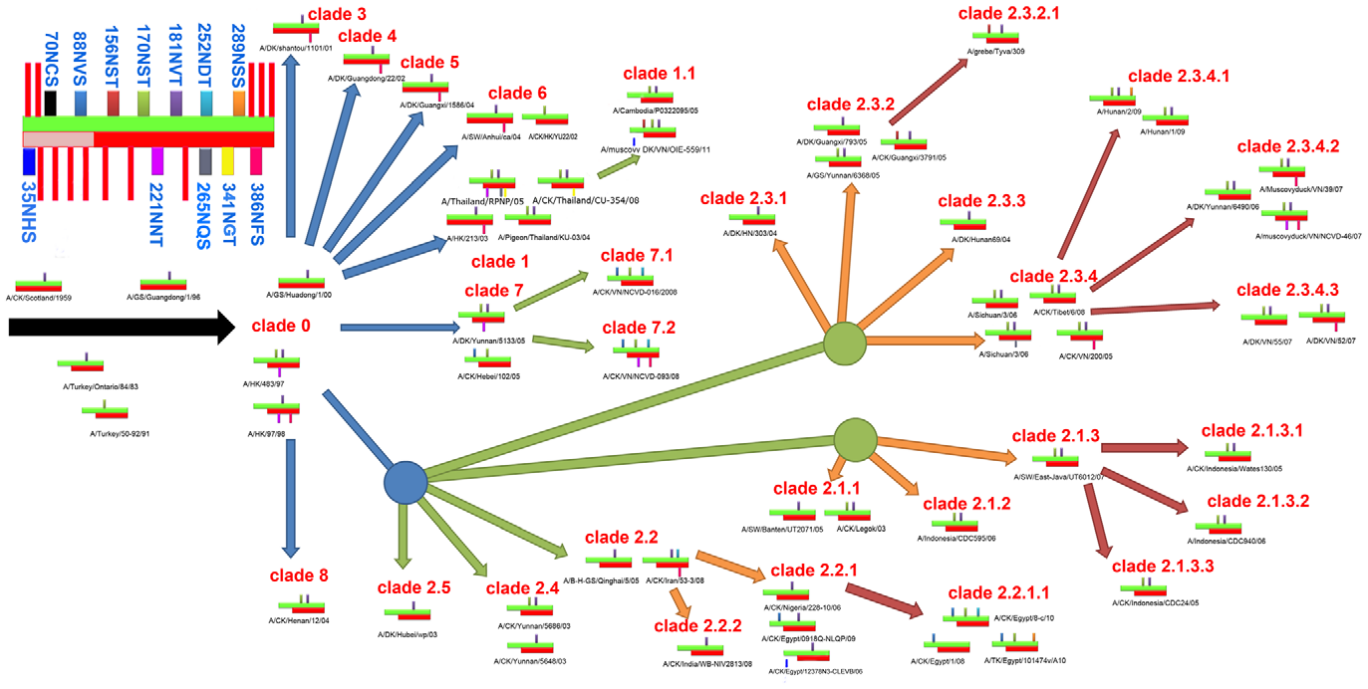
The co-evolution of glycosites between HA and NA in H5N1 virus. A schematic of diverse glycosites in HA and NA is shown in the top left corner. The green and red crossbands represent HA and NA, respectively, and the lightly colored area in NA is the stem domain. The negligible and highly conserved glycosites are shown in the red strip, while the remainder are labeled in cyan text. All the first-, second-, third- and fourth-order clades are shown with blue, green, orange and crimson arrows, respectively.

## Materials and Methods

### Protein sequence data from all subtypes of HA and NA

The amino acid sequences of HA and NA were obtained from the NCBI (National Center for Biotechnology Information) Influenza Resource (http://www.ncbi.nlm.nih.gov/genomes/FLU, accessed 15th March 2012) [5]. To fully understand the distributed regularity of the glycosites in each subtype, we downloaded the 29 sets of HA and NA with the customized definition “>{serotype} {strain} {segname}” using the following combinations: H1Nx∼H17Nx and HyN1∼HyN10 (where x and y represent “any” by default), in addition to the HA and NA from the H5N1 virus.

**Figure 5:**
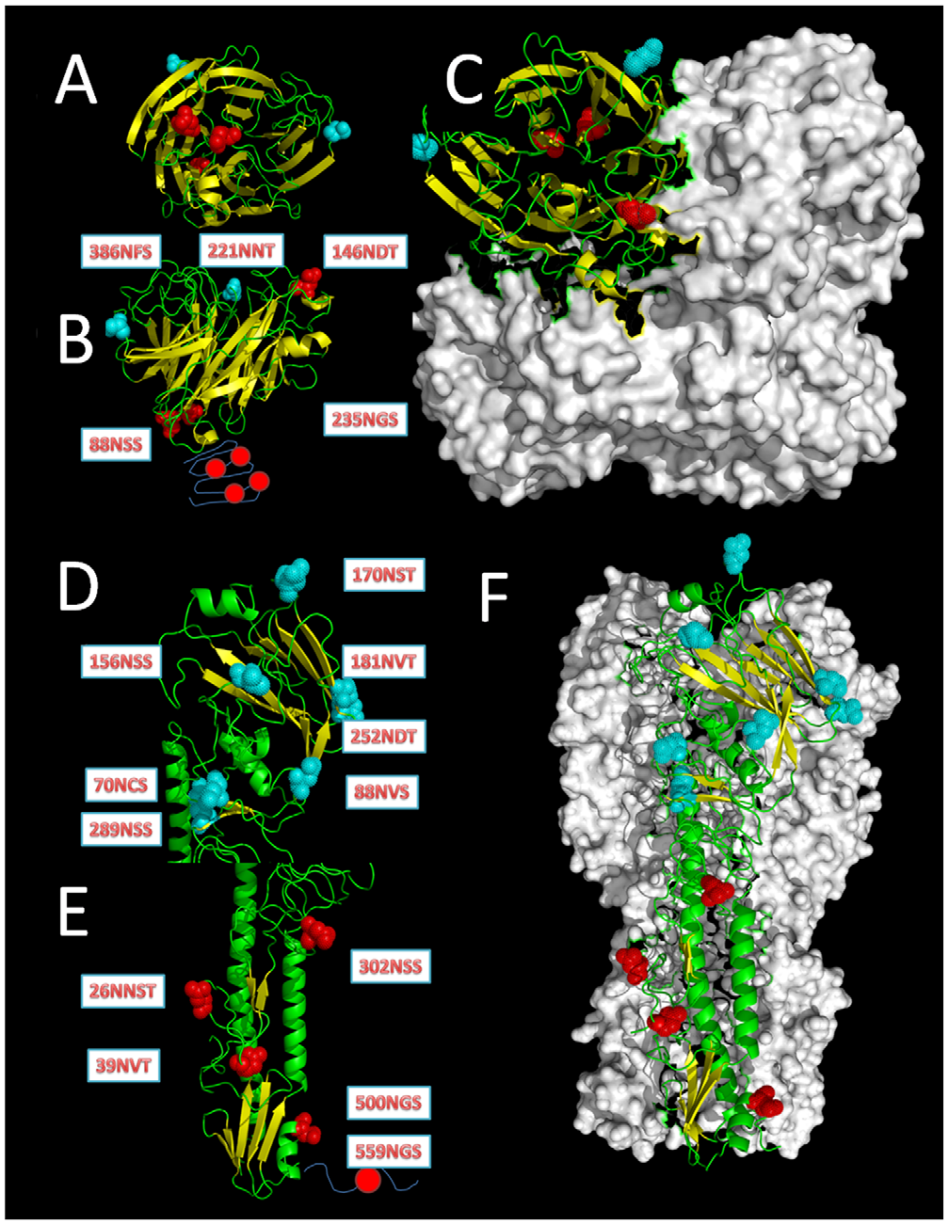
The full NAs and HAs structure and the distribution of glycosites. Those most conserved glycosites (>95%) are shown in red spheres, while the remainder are shown in cyan spheres. (A) The bottom view of the global domain of the NA monomer. (B) The side view of a NA monomer, the deletion of the stem domain would decrease four Glycosites. (C) The top view of the NA tetramer. (D) The side view of the global domain in the HA monomer. (E) The side view of the stem domain in the HA monomer, the 559NGS glycosite in the fusion peptide domain cannot be seen due to limited data. (F) The side view of the HA trimer.

All the sequence alignments of the various HAs and NAs were performed using Clustal 2.0. Repetitive, incomplete and mixed sequences were removed. For the purpose of convenience, one representative sequence with the generally longest length from each subtype was chosen for further description (Table S1 and S2).

**Figure 6:**
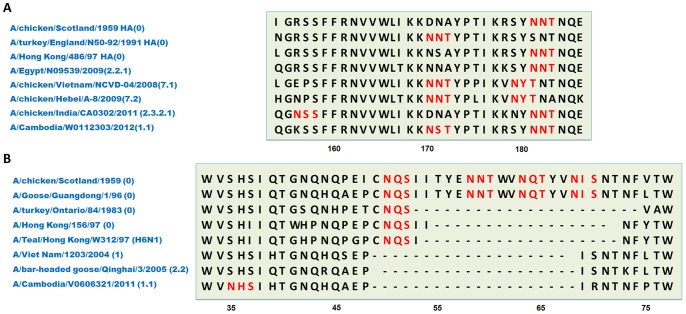
The alignment of two glycosylated regions in H5N1 HA and NA sequences. (A) The viruses with glycosylated and non-glycosylated 170N and 181N, which are the key glycosites in H5N1 HA, co-existed even before 1997. The nearby glycosites caused by “antigenic drift” were the “179NYT/181NNT”. The conserved 181NNT glycosite have been replaced by the 179NYT in parts of clade 7 viruses since 2005 (36 records in H5 set). Most viruses in clade 2.2 and clade 2.3.2 displayed the deficiency of 158N glycosite, while clade 1.1 and clade 2.3.4 exhibited the conservation of the 158N glycosite. (B) The deletion of the stalk domain in NA was considered as the characteristic of HPAI. Actually, the viruses with the missing and complete stalk domain co-existed even before 1997. The patterns of stalk domain deletion have two stages, with difference in 50NQS.

**Table 1:** The conservative rate of glycosites in the HA of H5N1.

Glycosite	Conservative rate	Glycosite	Conservative rate
26NNST	99.97%	39NVT	99.83%
70NCS	0.22%	88NVS	4.66%
156NSS	6.92%	170NST	46.58%
181NVT	95.29%	252NDT	3.99%
289NSS	2.10%	302NSS	99.41%
500NGS	99.47%	559NGS	99.53%

To assess the relationship between the distributed regularity of the glycosites in the envelope glycoproteins and the evolutionary position of various HAs and NAs, firstly, the preliminary trees for each HA and NA subtype were constructed using all available sequences; secondly ten representative sequences from discrepant clades, with consideration of host source and areas, were picked up (excluding H14, H17 and N10 due to limited records). Finally, the phylogenetic trees of HA and NA were constructed by these hundreds of sequences (File S1). The phylogenetic trees of the HA and NA were constructed by MEGA5.05 with N-J methods and a p-distance model with a bootstrap value of 1000 [41], [42].

**Table 2:** The conservative rate of glycosites in the NA of H5N1.

Glycosite	Conservative rate	Glycosite	Conservative rate
35NHS	1.39%	50NQS*	97.64%
58NNT*	100%	63NQT*	100%
68NIS*	96.30%	88NSS	99.09%
146NGT	98.54%	221NNT	0.84%
235NGS	99.34%	265NQS	0.15%
341NGT	0.88%	386NFS	10.72%

The asterisk represents those sites that were only calculated in the full stem domain.

### Prediction and statistical analysis of potential glycosites

As is known, N-X-T/S (X cannot be a proline) is the glycosite motif. Although some researchers have concluded that not all the potential glycosites would be glycosylated in mature glycoproteins, Kelley WM *et al*. argued that the 14-sugar glycans would be transferred to all the glycosites in nascent proteins and be truncated and modified in the subsequent process [42].

Due to the fixed pattern of glycosite, many programs and software provide the prediction of glycosites [43], such as the “NetNGlyc 1.0 Server” (www.cbs.dtu.dk/services/NetNGlyc). By submitting the alignment files to a prediction server, we obtained a series scores for the potential glycosites. In consideration of experimental error, those occasional glycosites, which may result from the genomic sequencing or translated by different genetic codes, were excluded. For example, the 429NLS in the HA of A/duck/Hunan/3315/2006(H5N1) only appears in H5 set once, and it is also embedded inside of HA trimer [44]. Statistics of the position, pattern and the levels of conservation in various subtypes were used for further discussion (Table S1, File S2).

### The comparison of 3D structures of HA and NA

The available structures of HA and NA would help us to explore the distinctive function of various glycosites. Therefore, we collected the structures of various HA or NA subtypes from the PDB (Protein Data Bank, http://www.rcsb.org/pdb/home/home.do). To obtain the whole multimer, these monomer structures were processed with a VMD 1.9 transformation matrix using a Tcl script [45]. The code for each representative structure of the various subtypes of HA and NA and the corresponding source of IVs are shown in Table S2.

Most coordinate files of envelope glycoproteins are obtained from X-ray crystallography or NMR. Furthermore, complete larger glycans are too ﬂexible to yield sufficient electron density [46]. Isolation and purification of membrane glycoproteins by particular enzyme treatments lead to the lack of partial domains [47]. In summary, complete structures are unavailable. Most recorded structures of HAs are lacking the HA0 cleavage site and partial HA2; while those of the NAs are lacking the stem domain.

### Co-evolution of glycosites in the H5N1 virus

The H5N1 virus can be divided into 10 clades, according to the evolutionary position of the HAs defined by the WHO (World Health Organization) in 2008. These clades are numbered 0 to 9 [48], [49]. Clades 0∼2 are responsible for all the human infections, resulting in 359 deaths since 2003 [11]. Many clades have not been reported for the last four years, while the dominant H5N1 viruses are concentrated in clade 0∼2. Clade 2.1, clade 2.2 and clade 2.3 have further evolved into third- or fourth-order clades, and these newly formed viruses have become geographic strains.

For the purpose of investigating the co-evolution relationship between the glycosite patterns of HA and NA, we reconstructed a phylogeny tree of HAs and determined ten clades. When discrete monophyletic groups with a common node meet a bootstrap value of ≥60 at the clade-defining node and average percentage pairwise nucleotide distances between and within clades of >1.5% and <1.5%, respectively, they could split into the second-, third-, or even fourth-order clades [50]. The statistics of the glycosites of each HA, as well as the corresponding NA, were recorded according to their clades (Files S3, S4 and Figure S1).

## Results

### Newly emerging Glycosites of HA appeared in distinctive subclades

As shown in Figure 1A, the current 17 HA subtypes were concentrated in two evolutionary groups. One large group, represented by H1 and H5, contained H2, H6, H8, H9, H11, H12, H13 and H16; Another large group was represented by H3, and contained H4, H7, H10, H14 and H15. Although each subtype of HA had a lower similarity to each other and the distribution of the glycosites differed in sequential numbering, we found that the sequence alignment of all HA subtypes indicated that partial glycosites appeared in similar domains. Furthermore, the structure alignment of available HAs also showed that various HA subtypes had a highly conserved structure, and the distribution of glycosites was also regular (Figure S2).

By analyzing the position of glycosites and their conservative rates, we conclude that two types of glycosites appear in HAs: one with a high level of conservation in all HA subtypes and another with various conservative rates in different HA subtypes. Two highly conserved glycosites are located near the HA0 cleavage site (e.g., the 27NNST in H1 or the 30NGT in H8) and the fusion peptide of the HA2 (e.g., the 498NGT in H1 or 500NGS in H5) respectively in all subtypes, and these two glycosites play necessary role in viral life cycle for protecting the HA0 cleavage sites and fusion peptide [34], [51]. In addition, another highly conserved glycosite appears at C-terminal part of the HA1 sequence, which is near the connection of the global and stalk domains, except in H7 and H15 sequences [52]. Three to ten characteristic glycosites were distinctive in each subtype. Their conservative rates were influenced by different internal evolution branches, ranked from 0.5% (e.g., 292NGS with 2.85% in H3) to 100% (e.g., 38NGT with 99.94% in H3), distributed mainly in the global domain. It is worth mentioning that some highly conserved glycosites were near to cysteines (Figure 1). Kozlov *et al*. hypothesized that ERp53, which is involved in the formation of disulfide bonds during the folding of nascent proteins, would form a complex with calreticulin/calnexin, which depends on precursor N-glycans [53].

One most obvious characteristics of glycosites was that the increasing samples or cross-species reports would lead to more glycosites. There are many factors contributing to the existence of numerous glycosites in H1, H3 and H5 subtypes. Because the RNA virus is rapidly mutating, long-term cross-species infections have resulted in the accumulation of adaptive mutations and glycosites. Due to the selection pressure of different host immune systems, a disadvantageous mutation would be eliminated while a virus with the gains and losses of glycosites would be conserved. For example, the HAs from avian H1N1 or H3N2 virus had less glycosites than those of the seasonal human H1N1 or H3N2 virus (e.g., 286NAS in A/Memphis/28/1983 (H1N1). Figure 2A and File 5S).

The H3 set had the most glycosites (16, as shown in File S6) among all HA subtypes, which were mainly contributed by H3N2 and H3N8 virus. The earliest H3 record had only one moderate conservative glycosite (79NCT, e.g. A/equine/Miami/1/1963 (H3N8)) except for six highly conserved glycosites, which is also conserved in most seasonal human H3N2 virus and mammalian H3N8 virus (e.g. A/Denmark/22/2011 (H3N2) or A/equine/Yokohama/aq79/2011 (H3N8)), but not in subsequent avian H3N1∼ H3N9 virus (e.g. A/duck/Zhejiang/5/2011 (H3N3) or A/duck/Saitama/2/2009 (H3N8). File S5). Over the last four decades, seasonal human H3N2 viruses have gradually acquired additional glycosites within the globular HA1 [37], [40]. The occurrence of 294NSS has increased the glycosite numbers to 13 in parts of current HA of H3N2 virus from 2010 (A/Singapore/GP5/2011(H3N2). File S6).

Not all the glycosites shared the same numbering in one subtype. For this reason, we chose a representative sequence for each subtype (Table S1). Most glycosites were influenced by the deletion or insertion of the mutants in the upstream sequence, which is also known as “antigenic drift” [54]. In the HAs of the H1N1 or H5N1 viruses, some nearby glycosites were determined by “antigenic shift” [55]. In the H1 subtypes, the 144NVT glycosite from the seasonal H1N1 flu, which were isolated from 1940s to 1980s (e.g., A/Memphis/1/1984 (H1N1)), was replaced by the 142NHT glycosite after the 1990s (e.g., A/California/04/2007 (H1N1)). Additionally, no such glycosylation appeared in those H1N1 viruses isolated from swine H1N1 flu (e.g., A/swine/Guangdong/1604/2010 (H1N1)), Spanish flu (e.g., A/South Carolina/1/1918 (H1N1)) and the 2009 influenza pandemic (H1N1) (e.g., A/Mexico city/CIA1/2009 (H1N1)). It has been partially shown that vaccination of mice with the 1918 influenza strain protected against subsequent lethal infection by the 2009 virus; however, the 1918 strain did not protect against the seasonal H1N1 flu [56]. It is interesting to note that the migration of the 179NKS to 177NLS tracks with these effects. Almost all of the 179NKS glycosites identified were centralized in swine-origin influenza viruses (S-OIVs, e.g., A/Swine/Guandong/1604/2010 (H1N1)) and parts of the 2009 influeza pandemic H1N1 virus (e.g., A/Mexico City/014/2009 (H1N1)), while the 177NLS glycosite was mainly found in the swine or human H1N2 viruses (e.g., A/New York/481/2003 (H1N2)) and most seasonal flu viruses (e.g., A/California/04/2007 (H1N1)). These date further confirm that the HA of the 2009 influenza pandemic (H1N1) originated from S-OIV, not previous seasonal virus [57]. Similar positional conversion of the glycosites in H5N1 caused by “antigenic drift” was the “179NYT/181NNT”. The conserved 181NNT glycosites have been replaced by the 179NYT in parts of clade 7 viruses since 2005. It reminds us that although these glycosites were adjacent, their N-glycans would shield different antigenic sites, which would provide some suggestions for the development of influenza vaccine.

Moreover, in the smaller cluster of H7, H10 and H15, one highly conserved glycosite appeared in the long a-helix of HA2 (e.g., 431NWT in A/turkey/Chile/4418/02 (H7N3)). This uncommon glycosite requires further investigation (File S1).

### The glycosylation of NA is affected by the deletion of the stalk domain

It can be observed from Figure 1B that N10 is highly divergent from other subtypes [4]. The remainder of the current 9 NA subtypes are concentrated into two evolutionary groups: one group was represented by N2 and contains N3, N6, N7, and N9; and another group contains N1, N4, N5, and N8.

The glycosites of NA can be divided into two types according to the distributed region: two to four highly conserved glycosites are located in the stalk domain in each subtype; two conserved glycosites and most middle-low conserved glycosites are mainly located in the global domain, which are near the tip of NA, the connection of the global and stalk domains, or the antigenic sites. The glycosite of 146N is conservative in all NA subtypes (e.g., 146NDT in A/Boston/20/2008(H3N2), 144NGT in A/duck/Taiwan/4201/99(H7N7) or 146NGT in /Viet Nam/1203/2004(H5N1)). It has been shown that the N-glycan at this glycosite affects NA enzymatic activity, causing a 20-fold decrease in activity [58]. Similar to the description of HAs, a large number of H1N1, H3N2, H5N1, H7N2 and H9N2 viruses have accumulated numerous glycosites in N1 and N2, especially in the global domain, mainly participating in immune evasion. Moreover, one conserved glycosite, 12NTT (Conservative rate: 93%, e.g., A/turkey/Italy/3807/2004 (H7N3)), located in the transmembrane domain in N3 and N10, requires further investigation [59].

The significance of the conserved glycosites in the stalk domain was providing the N-glycans to avoid the cleavage by host enzyme (e.g., Trypsin) [60], [61]. The variance of the glycosites was closely related to the deletion of the stalk domain. Although the three-dimensional structure of stalk domain has not been determined yet, it is speculated that the presence of an α-helix motif in the uncrystallized structure has also been provided by cryoelectron microscopy [62]. Wagner *et al*. believed that a longer stem domain would enhance the replication capacity of the virus, while the deletion of the stem domain would decrease the enzymatic activity of NA [63]. Various subtypes had stalk domain deletions of 3 to 24 residues, except for N4, N8, N9 and N10.

The numbers of deletions were also distinctive across different combinations of IVs and even within one subtype, such as the N2 subtype. Generally, no deletions were found in the NA of the H3N2 virus; deletion of 3 residues and one corresponding glycosite with the pattern “E-R-61N-3-64T-V-H” (meaning 3 residues missing between 61N and 64T, e.g., A/chicken/Zhejiang/611/2011 (H9N2)), appeared in most of the NA subtypes of the H9N2 virus. A similar deletion of 20 residues and two glycosites (I-E-60R-20-80N-I-I) appeared in most NA subtypes of the H6N2 virus (e.g., A/duck/Fujian/3193/2005 (H6N2)). The deletion of 16 residues and two glycosites as “C-E-55P-16-72T-T-E” were a distinctive part of the H7N2 virus (e.g., A/unknown/New York/19501-5/2006 (H7N2)). Parts of the H5N2 virus were characterized by the deletion of 20 residues and two glycosites (“R-N-62I-20-83G-Y-R”, e.g., A/chicken/Ibaraki/3/2005 (H5N2)). Moreover, in some avian H2N2 viruses, the deletion of 22 residues resulted in the loss of two glycosites; however, a new glycosite appeared in the newly created sequences (“P-A-47N-22-70N-T-V”, e.g., A/chicken/New York/Sg-00300/1997 (H2N2)). In all, the diversity of deletions in the stalk domain indicates that the glycosylation pattern of HA and NA has a complex relationship.

### The co-evolutionary glycosite pattern in the H5N1 virus

Globally, the researchers have been paying attention to the highly pathogenic avian influenza (HPAI) since the first human death caused directly by avian H5N1 virus in 1997. It is generally considered that the HPAI viruses were characterized by polybasic residues in the HA0 cleavage site in HA and the deletion of the stalk domain in NA [64]. However, these characteristics had been reported even before 1990s. The earliest H5N1 virus, “A/chicken/Scotland/1959 (H5N1)”, had four continuous basic residues in the HA0 cleavage site (compared to 5∼6 continuous basic residues in most common H5N1 HAs); the stalked deletion of NA also existed in A/turkey/Ontario/84/1983 (H5N1). The records of H5N1 virus have increased rapidly since 2003. Since then, a number of new clades and subclades have emerged and resulted in various new glycosites (Figure 3A and 3B). The percentage of unconserved glycosites in HA also increased and diversified rapidly after 2003, meanwhile the percentage of unconserved glycosites in NA reduced gradually, regardless of those highly-conserved glycosites (Figure 3C and 3D).

Both twelve glycosites were found in the HA and NA of the H5N1 virus, shown in the Tables 1 and 2 and Files S3 and S4.

Most H5N1 viruses were grouped into clade 0 before it appeared in Hong Kong again in 2003. Various patterns of the glycosites in HA and NA had co-existed in these original viruses. These original NAs contained the known glycosites, including four highly conserved glycosites in the stalk domain and seven in the global domain (except the occasional 341NGT which only appeared in clade 1 and Thailand records during 2004∼2010, e.g., A/chicken/Thailand/CU-354/2008 (H5N1)). In contrast, six highly conserved glycosites together with 170NST exist widely in clade 0. Since 2003, the WHO has recorded a three-wave epidemic of H5N1, which resulted in hundreds of deaths and huge economic losses [11]. Until recently, the glycosite patterns were highly conserved in all the avian clades except for clade 7 (e.g., clade 3, 4, 5, 6, 8 and 9), as shown in Figure 4 and Figure S2.

Most currently recorded H5N1 viruses were concentrated in the fourth-order clades. During this decade, there have been increasing human infections and new glycosite patterns of HA and NA. It has been reported that the H5N1 virus of clade 2.2 was involved in the outbreak that occurred among the migratory bird population near Qinghai Lake in 2005. Since then clade 2.2 spread westward. This resulted in a number of deaths of wild birds in Europe [65]. In 2006, the H5N1 virus appeared in Africa for the first time, followed by hundreds of mortally infected humans in Egypt, Nigeria and Djibouti [66]. The most recent H5N1 virus isolated from North Africa belongs to clade 2.2.1.1, along with one novel glycosite 88NVS. In addition, most viruses in clade 2.2, except for clade 2.2.1.1, lack the 170N glycosite created by the T172A mutation. Wang *et al*. concluded that the lack of 170NST would enhance the HA affinity for SA receptors, especially to SAα2-6Gal sialoglycans, which could be one reason for the propensity of clade 2.2.1 to infect humans [67], compared with only three conserved glycosites in the NA of clade 2.2.1.1 (Figure 4, Files S3).

Parts of the H5N1 virus isolated from China and Vietnam belong to clade 2.3.4, with conserved glycosites in HA, but not in NA. In contrast, another dominant virus from clade 2.3.2 featured the loss of 170NST in HA and stable glycosites in NA. Within the current clade 2.3.2.1, a new glycosite has appeared, “156NSS”, which co-existed with 88NVS near the antigen sites (Figure 5D, Files S3).

The 181NVT glycosite, which is located at the apical β-folding of HA, is conserved in all human IVs (e.g., A/Anhui/1/2007 (H5N1)). Previous statistics have indicated that this glycosite has a lower level of conservation in avian IVs (e.g., A/chicken/Vietnam/NCVD-093/2008 (H5N1)). The results of the molecular dynamics simulation indicated that the α2-3-sialoglycans adopted a straight-like and outward topology structure while the α2-6-sialoglycans were fishhook-like and inward; therefore, we inferred that the that the deficiency of the glycosite would benefit the binding of SAα2-3Gal sialoglycans [68]. Actually, all the viruses that had deficiencies of the 181N glycosite were isolated from the avian host, which were concentrated in clades 7.1, 7.2 and 2.2.1.1 (Figure 6A, Files S3).

Interestingly, the deletion of the stalk domain in H5N1 NA is variable. As the first human death reported in 1997, most H5N1 viruses that belonged to clade 0 and isolated in Hong Kong were characterized by “N-Q-S-I-54I-18-73N-F-Y”, which remained a glycosite: 50NQS. Although previous studies conjectured that the H6N1 virus (e.g., A/Teal/Hong Kong/W312/97 (H6N1)) was the donor of the NA gene in 1997 HPAI virus [69], [70]; however, similar motif could be found even in 1983. Since 2000, the most common pattern of “A-E-48P-20-69I-S-N” with four glycosites missing has dominated in the H5N1 NAs (Figure 6B, File S4).

## Discussion

There is one kind of glycoprotein that participates in the recognition and membrane fusion in most virus envelopes, such as the spike (S) protein in the SARS virus, the gp160 in HIV and HA in IVs [71], [72]. These glycoproteins can bind to one specific glycan structure which is known as the lectin or GBP. Other viral glycoproteins, such as HN in the Newcastle Disease Virus or NA in IVs, function as the exoglycosidase in the release of virus particles [73].

The IVs have an innate capacity for high mutation rates because they are RNA viruses. Humans are under continuous attacks by newly emerging IVs which constantly undergo “antigenic drift” and “antigenic shift”. Accumulation of substantial sequences and 3D coordinates of IV proteins have provided the ideal tools for the investigation of how mutations affect transfaunation, vaccine design and drug-resistance [74], [75].

N-glycosylation not only influences the folding and secretion of glycoproteins such as HA and NA but also provides the same glycans (similar to the host's own glycans) to escape the host's immune system. As the key modification of biological significance in viral glycoproteins, we found the glycosites in 17 known HA subtypes and 10 known NA subtypes that have complex characteristics. In general, two highly conserved glycosites near the HA0 cleavage site and fusion peptide site may maintain the basic function of HA; these sites were seldom absent in the HA subtypes. More glycosites were identified in the global domain or in the connection of global and stalk domains, along with long-term and large-scale epidemics in several of the subtypes. The distributional regularity of the glycosites in the NA subtypes is also complex; two to four glycosites located in the stalk domain are highly conserved in various subtypes, and are affected by the deletion of the stalk domain. Another highly conserved glycosite was found at the tip of tetramer NA in all subtypes. Other glycosites were found to be mainly concentrated in the global domain, which surrounds the antigenic sites.

The HA or NA subtypes exhibited low similarities of amino acid sequences in all subtypes while maintaining identical structures, which revealed that the functions of various HA or NA subtypes remain conserved. Notably, some of the glycosites near the cysteines were found to also be conserved. The cysteines take the main role in stabilizing the tertiary structure; the conservation verifies that cysteines and N-glycans played an important role in the protein folding and quality control.

We have further investigated the H5N1 virus to elaborate the collaborative relationship of glycosites in HA and NA. Five highly conserved glycosites in HA had existed before the H5N1 virus first crossed species barriers and infected humans, as well as two additional glycosites, 181NVT and 170NST. Since 2003, the H5N1 virus has exhibited a rapid evolutionary dynamic. Under the selection pressure of different hosts and antigenic drift, the glycosite pattern of current H5N1 viruses in different geographical locations has been distinctive: the HA in clade 2.2.1.1, isolated from Egypt, lacked the 181N glycosite but had added the 88NVS glycosite. In addition, the HA in clade 2.3.2.1, isolated from China or Vietnam, lacked the 170N glycosite but had added the 152N glycosite. In contrast, as the glycosites in HA became more diverse in the H5N1 virus, the glycosylation in NA was impaired by a decreasing number of glycosites. The NAs of current H5N1 viruses lack a stalk domain and the four corresponding glycosites; except the four high conserved glycosites in the global domain, others glycosites have rarely been identified.

The envelope glycoproteins, which play a crucial role in virus recognition, invasion and spread. The analysis of the glycosites in HAs and NAs has provided basic information for vaccine design, host selection and changing virulence. However, infection is a complex process; the alternation of glycosites and glycan shapes may affect the functions of glycoproteins. In addition, there are other mutations also worthy of further consideration, such as the E627K in the PB2 protein that enhances the avian viral replication capacity in mammalian cells [76], [77]. Drug resistance is also related to viral proteins, such as the M2 protein [78]. Thus, to prevent the next influenza pandemics, more research needs to be done.

## Supporting Information

Figure S1 **The N-J trees of H5N1 HA.** The phylogenetic tree was inferred from protein sequences by the Neighbor-Joining method and rooted using A/chicken/Scotland/1959. Estimates of the statistical significance of the phylogenies were calculated by performing 1,000 bootstrap replicates. The clades classified by the WHO are shown as colored bars.
(TIF)

Figure S2 **The superposition of crystal structures from various HA and NA subtypes.** The red, green, blue and yellow color represent the H1, H3, H5 and H7 in HAs or N1, N2, N8 and N9 in NA respectively. (A) The side view of the global domain of NA. (B) The bottom view of the global domain of NA. (C) The top view of the global domain of NA (D,E). The side view of the HA trimer.
(TIF)

File S1 **The analytic alignment file of 146 representative HA and 93 representative NA sequences.**
(XLSX)

File S2 **The conservative rate of glycosites in 17HA and 10 NA subtypes.** The asterisk represents those sites that were only calculated in the full stem domain.
(XLS)

File S3 **The analytic alignment file containing the 3576 protein sequences of H5N1 HA.** As is shown in the output file, all of the glycosites are marked in red.
(XLSX)

File S4 **The analytic alignment file containing the 2732 protein sequences of H5N1 NA.** As is shown in the output file, all of the glycosites are marked in red.
(XLSX)

File S5 **The analytic alignment file containing the 5973 protein sequences of H3 HA.** As is shown in the output file, the red, green and blue color represent the levels of conservation of “>95%”, “5%∼95%” and “<5%”, respectively.
(ZIP)

File S6 **The N-J trees of H3 HA.** The phylogenetic tree was inferred from H3 HA sequences by the N-J method and rooted using A/equine/Miami/1/1963(H3N8). Estimates of the statistical significance of the phylogenies were calculated by performing 1,000 bootstrap replicates. 633 representative H3 HA sequences were chosed in consideration of the age, hosts, areas, as well as the known HA sequences from H3N1 and H3N3∼H3N7 viruses. Those unconserved glycosites are labeled (conservative rate <95%).
(PDFS)

Table S1 **Subtypes of HAs/NAs and their representative strains.**
(DOC)

Table S2 **Representative IDs of HAs/NAs in the PDB and their strains.**
(DOC)
