# Supplementary figures and images for: The Evolutionary Pattern of Glycosylation Sites in Influenza Virus (H5N1) Hemagglutinin and Neuraminidase
Source: PLoS One. 2012 Nov 1;7(11):e49224. doi: 10.1371/journal.pone.0049224 (PMC3486865; doi:10.1371/journal.pone.0049224)

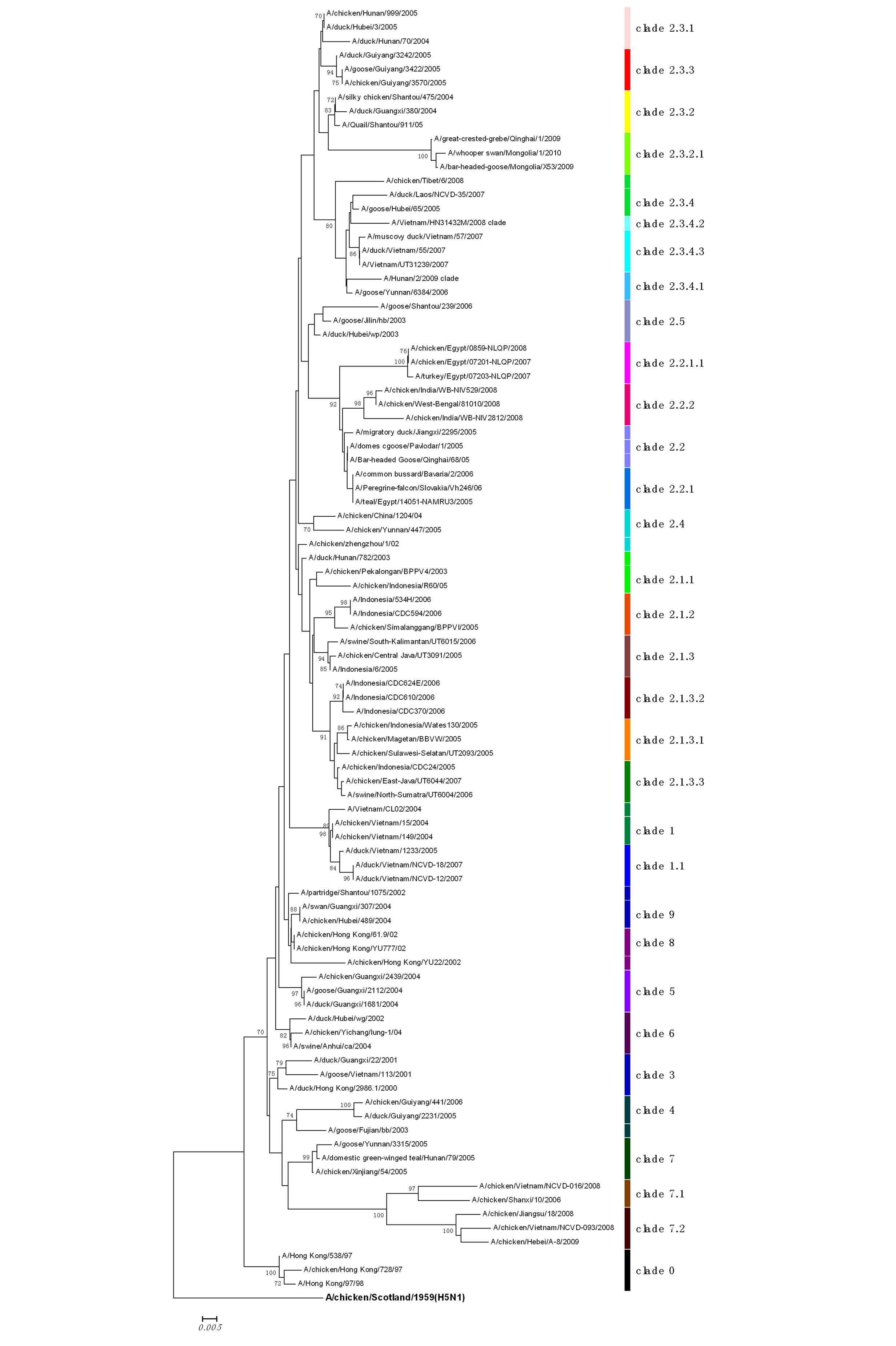

Supplement: Figure S1 — The N-J trees of H5N1 HA. The phylogenetic tree was inferred from protein sequences by the Neighbor-Joining method and rooted using A/chicken/Scotland/1959. Estimates of the statistical significance of the phylogenies were calculated by performing 1,000 bootstrap replicates. The clades classified by the WHO are shown as colored bars. (TIF) [file pone.0049224.s001.tif]

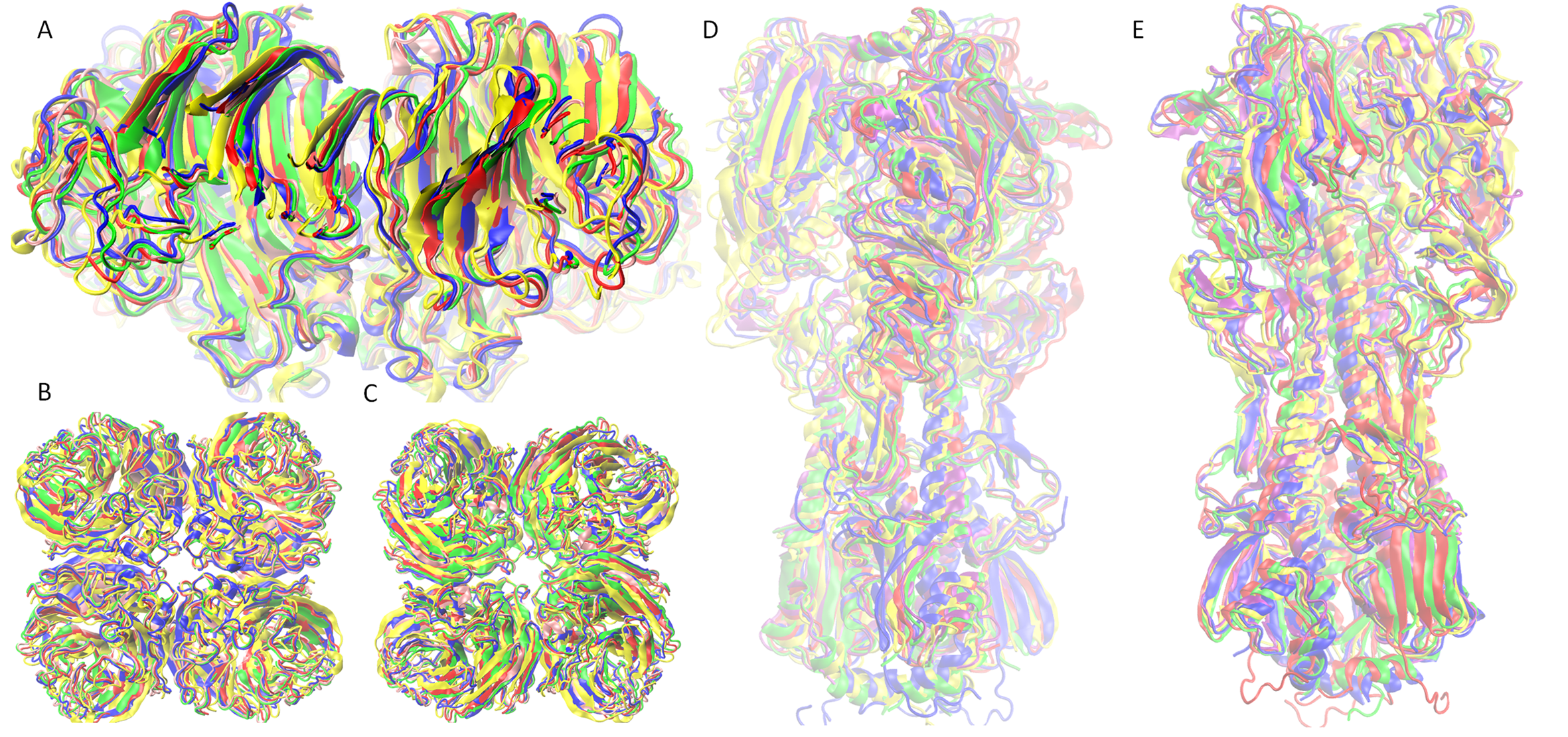

Supplement: Figure S2 — The superposition of crystal structures from various HA and NA subtypes. The red, green, blue and yellow color represent the H1, H3, H5 and H7 in HAs or N1, N2, N8 and N9 in NA respectively. (A) The side view of the global domain of NA. (B) The bottom view of the global domain of NA. (C) The top view of the global domain of NA (D,E). The side view of the HA trimer. (TIF) [file pone.0049224.s002.tif]
