# Supplementary material for: The Evolutionary Pattern of Glycosylation Sites in Influenza Virus (H5N1) Hemagglutinin and Neuraminidase
Source: PLoS One. 2012 Nov 1;7(11):e49224. doi: 10.1371/journal.pone.0049224 (PMC3486865; doi:10.1371/journal.pone.0049224)
Supplement: Table S1 — Subtypes of HAs/NAs and their representative strains. (DOC) [file pone.0049224.s009.doc]

**Subtypes of hemagglutinins and their representative strains**

| Subtype | Representative strain | Length | Sum. |
| --- | --- | --- | --- |
| H1 | A/Alagoas/96/2010 (H1N1) | 566 | 6480 |
| H2 | A/Japan/305/1957 (H2N2) | 562 | 222 |
| H3 | A/X-31(H3N2) | 566 | 5973 |
| H4 | A/northern shoveler/california/10024/2008 (H4N4) | 564 | 417 |
| H5 | A/duck/Germany/R338/06 568 (H5N1) | 568 | 3441 |
| H6 | A/duck/hunan/491/2005 (H6N2) | 566 | 604 |
| H7 | A/equine/Prague/2/1956 (H7N7) | 570 | 881 |
| H8 | A/duck/yangzhou/02/2005 (H8N4) | 566 | 63 |
| H9 | A/chicken/Guangdong/ZCY/2011 (H9N2) | 560 | 1321 |
| H10 | A/mallard/Alberta/209/2003 (H10N7) | 561 | 236 |
| H11 | A/shoveler/Netherlands/19/1999 (H11N9) | 565 | 186 |
| H12 | A/pintail duck/Alberta/49/2003 (H12N5) | 564 | 79 |
| H13 | A/duck/Siberia/272/1998 (H13N6) | 566 | 54 |
| H14 | A/mallard/Astrakhan/263/1982 (H14N5) | 568 | 3 |
| H15 | A/teal/Chany/7119/2008 (H15N4) | 570 | 15 |
| H16 | A/black-headed gull/Sweden/5/99 (H16N3) | 566 | 19 |
| H17 | A/ bat/Guatemala/060/2010(H17N10) | 564 | 3 |
| HA(H5N1) | A/duck/Germany/R338/06 568 (H5N1) | 568 | 2933 |

**Subtypes of Neuraminidases and their representative strains**

| Subtype | Representative strain | Length | Sum |
| --- | --- | --- | --- |
| N1 | A/Texas/UR06-0309/2007 (H1N1) | 470 | 11961 |
| N2 | A/Japan/305/1957 (H2N2) | 469 | 4651 |
| N3 | A/duck/PA/143585/2001 (H5N3) | 469 | 509 |
| N4 | A/duck/yangzhou/02/2005 (H8N4) | 470 | 124 |
| N5 | A/pintail duck/Alberta/49/2003 | 473 | 150 |
| N6 | A/duck/Siberia/272/1998 (H13N6) | 470 | 667 |
| N7 | A/equine/Prague/2/1956 (H7N7) | 471 | 248 |
| N8 | A/duck/Hunan/3748/2004 (H6N8) | 470 | 862 |
| N9 | A/shoveler/Netherlands/19/1999 (H11N9) | 470 | 225 |
| N10 | A/ bat/Guatemala/060/2010(H17N10) | 442 | 3 |
| NA(H5N1) | A/mallard/Wisconsin/2576/2009 (H5N1) | 469 | 2599 |
