# Supplementary material for: The Evolutionary Pattern of Glycosylation Sites in Influenza Virus (H5N1) Hemagglutinin and Neuraminidase
Source: PLoS One. 2012 Nov 1;7(11):e49224. doi: 10.1371/journal.pone.0049224 (PMC3486865; doi:10.1371/journal.pone.0049224)
Supplement: Table S2 — Representative IDs of HAs/NAs in the PDB and their strains. (DOC) [file pone.0049224.s010.doc]

**Representative IDs of hemagglutinins in PDB and their strains**

| Subtype | PDB code | Original strain |
| --- | --- | --- |
| H1 | 3AL4 | A/California/04/2009 (H1N1) |
| H2 | 3QQO | A/Japan/305/1957 (H2N2) |
| H3 | 1HGF | A/X-31 (H3N2) |
| H5 | 2IBX | A/Viet Nam/1194/2004 (H5N1) |
| H7 | 3M5J | A/Environment/New York/30732-1/2005 (H7N2) |
| H9 | 1JSH | A/Swine/Hong Kong/9/1998 (H9N2) |

**Representative IDs of Neuraminidases in PDB and their strains**

| Subtype | PDB code | Original strain |
| --- | --- | --- |
| N1 | 3TI4 | A/California/04/2009 (H1N1) |
| N1 | 2HU4 | A/Viet Nam/1203/2004 (H5N1) |
| N2 | 1ING | A/Tokyo/3/1967 (H2N2) |
| N2 | 2AEQ | A/Memphis/31/1998 (H3N2) |
| N4 | 2HTW | A/mink/Sweden/E12665/1984 (H10N4) |
| N5 | 3TI8 | A/duck/Alberta/60/1976 (H12N5) |
| N6 | 1VOZ | English Duck(UNKONW) |
| N8 | 2HTU | A/duck/Ukraine/1/1963 (H3N8) |
| N9 | 1NNB | A/tern/Australia/G70C/1975 (H11N9) |
