# Supplementary material for: The Evolutionary Pattern of Glycosylation Sites in Influenza Virus (H5N1) Hemagglutinin and Neuraminidase
Source: PLoS One. 2012 Nov 1;7(11):e49224. doi: 10.1371/journal.pone.0049224 (PMC3486865; doi:10.1371/journal.pone.0049224)

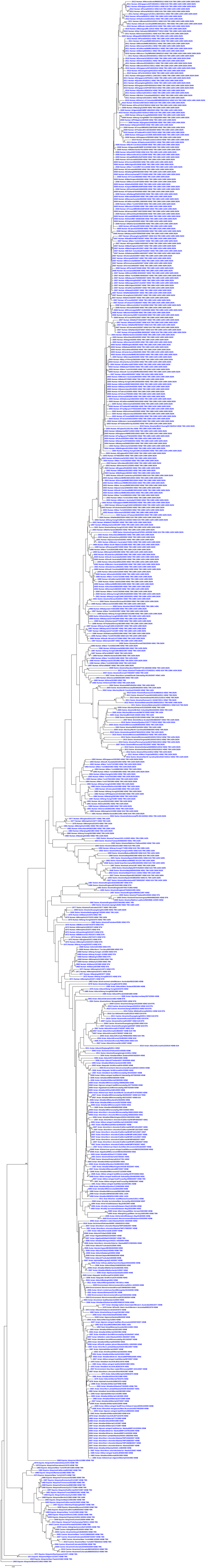

Supplement: File S6 — The N-J trees of H3 HA. The phylogenetic tree was inferred from H3 HA sequences by the N-J method and rooted using A/equine/Miami/1/1963(H3N8). Estimates of the statistical significance of the phylogenies were calculated by performing 1,000 bootstrap replicates. 633 representative H3 HA sequences were chosed in consideration of the age, hosts, areas, as well as the known HA sequences from H3N1 and H3N3∼H3N7 viruses. Those unconserved glycosites are labeled (conservative rate <95%). (PDFS) [file pone.0049224.s008.pdfs]
